# Impact of Three Different Serum Sources on Functional Properties of Equine Mesenchymal Stromal Cells

**DOI:** 10.3389/fvets.2021.634064

**Published:** 2021-04-30

**Authors:** Lynn Pezzanite, Lyndah Chow, Gregg Griffenhagen, Steven Dow, Laurie Goodrich

**Affiliations:** ^1^Department of Clinical Sciences, College of Veterinary Medicine and Biomedical Sciences, Colorado State University, Fort Collins, CO, United States; ^2^Department of Microbiology, Immunology and Pathology, College of Veterinary Medicine and Biomedical Sciences, Colorado State University, Fort Collins, CO, United States

**Keywords:** equine, mesenchymal stem cell, serum, fetal bovine serum, mesenchymal stromal cell

## Abstract

Culture and expansion of equine mesenchymal stromal cells (MSCs) are routinely performed using fetal bovine serum (FBS) as a source of growth factors, nutrients, and extracellular matrix proteins. However, the desire to minimize introduction of xenogeneic bovine proteins or pathogens and to standardize cellular products intended for clinical application has driven evaluation of alternatives to FBS. Replacement of FBS in culture for several days before administration has been proposed to reduce antigenicity and potentially prolong survival after injection. However, the functional consequences of MSC culture in different serum types have not been fully evaluated. The objective of this study was to compare the immunomodulatory and antibacterial properties of MSCs cultured in three serum sources: FBS or autologous or allogeneic equine serum. We hypothesized that continuous culture in FBS would generate MSCs with improved functionality compared to equine serum and that there would not be important differences between MSCs cultured in autologous vs. allogeneic equine serum. To address these questions, MSCs from three healthy donor horses were expanded in medium with FBS and then switched to culture in FBS or autologous or allogeneic equine serum for 72 h. The impact of this 72-h culture period in different sera on cell viability, cell doubling time, cell morphology, bactericidal capability, chondrogenic differentiation, and production of cytokines and antimicrobial peptides was assessed. Altering serum source did not affect cell viability or morphology. However, cells cultured in FBS had shorter cell doubling times and secreted more interleukin 4 (IL-4), IL-5, IL-17, RANTES, granulocyte–macrophage colony-stimulating factor, fibroblast growth factor 2, eotaxin, and antimicrobial peptide cathelicidin/LL-37 than cells cultured in either source of equine serum. Cells cultured in FBS also exhibited greater spontaneous bactericidal activity. Notably, significant differences in any of these parameters were not observed when autologous vs. allogeneic equine serum was used for cell culture. Chondrogenic differentiation was not different between different serum sources. These results indicate that MSC culture in FBS will generate more functional cells based on a number of parameters and that the theoretical risks of FBS use in MSC culture should be weighed against the loss of MSC function likely to be incurred from culture in equine serum.

## Introduction

Mesenchymal stromal cells (MSCs) derived from bone marrow, adipose, or blood tissues exert potent immunomodulatory and antibacterial activities, which renders them attractive as biological therapies for diverse conditions, including musculoskeletal injuries, wound healing, and bacterial infections (1–12). *In vitro* cell expansion of MSCs is required to obtain enough cells for clinical use, and a number of previous studies have evaluated the impact of tissue culture medium and serum source on relevant MSC properties (13). Culture and expansion of equine MSCs are routinely performed using fetal bovine serum (FBS) as a source of growth factors, nutrients, and extracellular matrix proteins. However, the use of FBS to expand equine MSCs has been linked to potential hypersensitivity reactions and the risk of introduction of viral or prion pathogens (14–17). The International Society for Cellular Therapy and several regulatory agencies have responded with position statements recommending the avoidance of FBS in MSC culture for clinical applications when possible and have called for a consensus on serum replacements in cell culture media (16–20).

Alternatives to FBS supplementation include autologous or allogeneic serum, purified recombinant or synthetic proteins, platelet lysate, and defined serum-free medium (16, 21–24). With regard to defined serum-free medium, multiple different commercial formulations are available, but all suffer from high cost, which renders their use for clinical studies cost-prohibitive in some situations. Therefore, species-matched serum (autologous or allogeneic) with conventional cell culture medium has been proposed as an alternative to FBS. The impact of serum source on MSC properties has been most fully explored with human MSCs, with conflicting results in terms of proliferation and differentiation, but few studies have described serum-free culture of MSCs from large animal veterinary species (13, 25–44). When comparing culture of human MSCs in FBS, human serum, or platelet lysate, Aldahmash et al. and Perez-Ilzarbe et al. found no difference in morphology or capacity for differentiation and proliferation growth rates, whereas Kuznetsov et al. reported FBS media culture resulted in greater proliferation and enhanced bone formation following *in vivo* transplantation (38, 39, 43). Schubert et al. compared serum-free culture of human and equine MSCs, demonstrating that culture of equine, but not human MSCs, in serum-free conditions resulted in altered morphology and variable proliferation and surface immunophenotype, which seemed to depend on the media lot (13). These findings emphasize that media formulations are specific for cell types and culture procedures and that development of media conditions should be optimized for MSCs from the animal species of interest. However, to date, the relative effects of FBS vs. equine autologous or allogeneic serum on the functional properties of equine MSCs have not been evaluated.

Therefore, the aims of this study were to compare relevant functional properties of equine MSCs cultured in medium with either FBS, equine autologous serum, or equine allogeneic serum. The functional properties evaluated included cell viability, proliferation rate, morphology, concentration of cytokines and antimicrobial peptides in MSC-conditioned medium (MSC-CM), bactericidal activity, and chondrogenic differentiation potential. We hypothesized that cells would have greater functionality when cultured in FBS containing medium and that there would be no significant differences in functionality when culture in autologous and allogeneic equine serum was compared.

## Methods

### Horses

**Figure 1:**
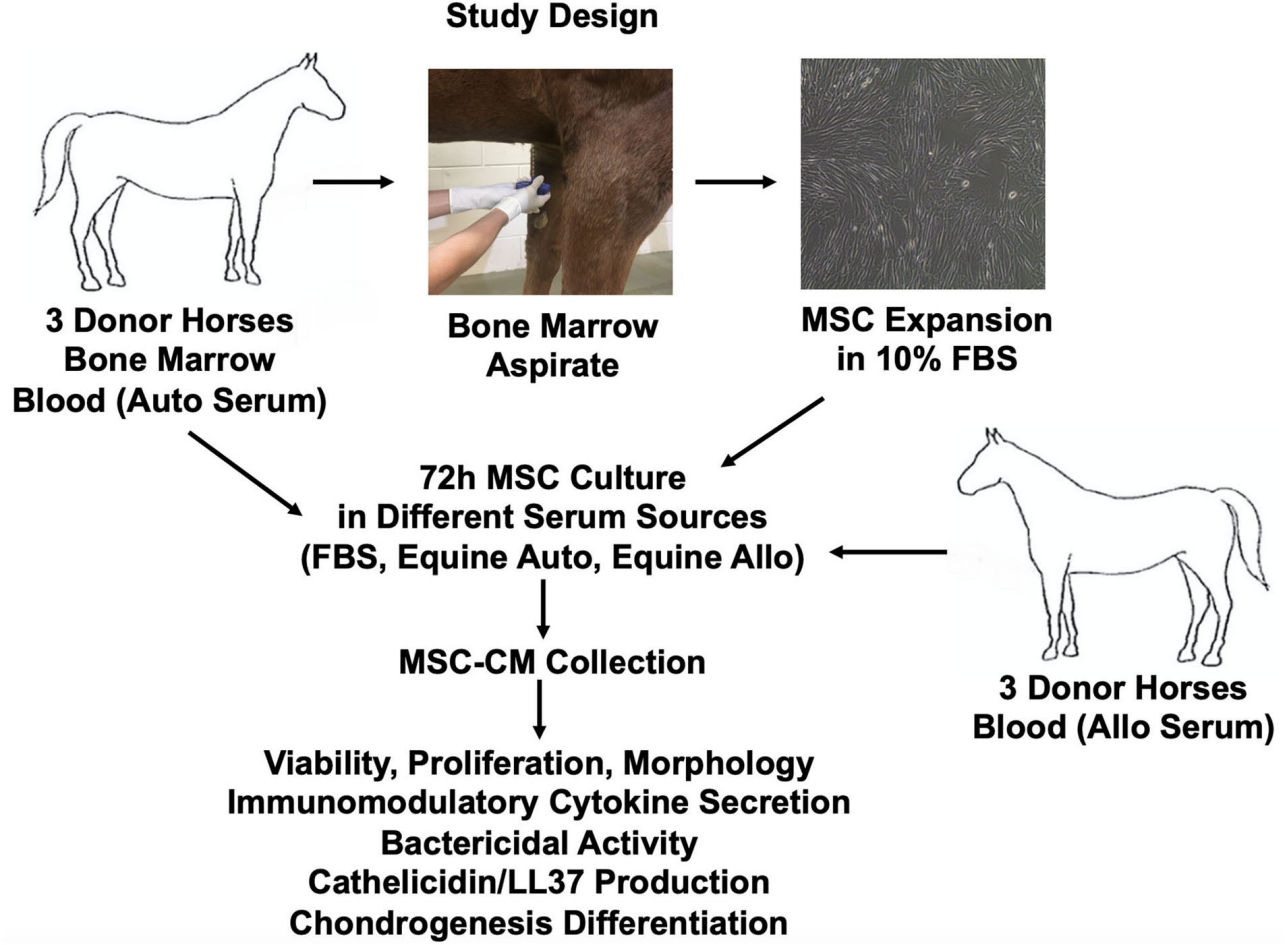
Schematic overview of study design and methods used. Allo, allogeneic; Auto, autologous; FBS, fetal bovine serum.

Schematic overview of study design and methods used is provided in Figure 1. Six healthy 2–3-year-old Quarter Horse research horses (three geldings, three mares) were tissue donors in this study. All horses were part of the university-owned herd at Colorado State University, and studies were approved by the Institutional Animal Care and Use Committee (protocol 1101). All horses were determined healthy by physical examination and blood work (complete blood count, diagnostic panel). Three horses were used as donors of bone marrow aspirate and autologous serum, and three different horses were used as donors of allogeneic serum.

### Tissue Collection and Cell Culture

To collect bone marrow aspirate, the sternum of three donor horses (Quarter horses, two geldings, and one mare; aged 2.5, 2.5, and 3 years) was clipped and aseptically prepared in routine fashion. Bone marrow aspirate (5 mL) was obtained using 11-gauge Jamshidi into a syringe containing 1 mL heparin (10,000 IU). Bone marrow aspirate was plated on 75-cm^2^ plates in complete supplemented growth medium [Dulbecco modified eagle medium (DMEM), 10% FBS, penicillin (100 U/mL), streptomycin (100 μg/mL), 1 mol/L HEPES]. The MSCs were expanded in culture and then frozen at 5 × 10^6^/mL in freeze media (90% FBS, 10% dimethyl sulfoxide) in liquid nitrogen vapor phase until further use. All MSCs used for *in vitro* studies were evaluated for surface phenotype and found to be CD44^+^CD90^+^ and CD34^−^CD45^−^, using equine cross-reactive antibodies as previously described (45) and in accordance with the International Society for Cellular Therapy minimal criteria for defining MSCs (46).

To obtain equine serum for use in cell culture, whole blood was collected from all six donor horses (three autologous and three allogeneic to MSCs cultured) into red-top blood tubes lacking anticoagulant. Blood tubes were spun at 1,800 relative centrifugal force for 10 min, and serum was removed, heat-inactivated (heated 30 min at 56°C with mixing), sterile filtered, aliquoted in 1-mL aliquots, and frozen at −80°C for later use. Serum from the same three donors as was used for bone marrow aspirate donated “autologous serum” in all experiments. Pooled serum from three additional donors (Quarter horses, one gelding, and two mares; aged 2, 3, and 3 years) was used as “allogeneic serum” in all experiments.

### Viability, Proliferation, and Morphology

Cells were thawed quickly in a 37°C water bath, recovered, and expanded for at least 48 h under standard incubation conditions (37°C with 5% CO_2_) in complete growth media with 10% FBS supplementation. Cells were then transitioned to growth media containing either FBS or autologous or allogeneic equine serum, as subsequently described. Cells were used between passage 1 and 3 for all experiments described. Plates and flasks containing MSCs were stored in the incubator (37°C with 5% CO_2_) following serum treatment for all experiments.

To assess cell viability following culture in media containing various serum sources, MSCs from three individual horse donors were plated at 50,000 cells/well on 24-well plates in growth media containing either 10% FBS or autologous or allogeneic equine serum. Cell viability was assessed using trypan blue dye exclusion staining to determine percentage of live cells following 24, 48, and 72 h in culture using an automated cell counter (Nexcelom; Bioscience Cellometer Auto T4). Experiments were performed in duplicate for all three donors, with each donor assessed in triplicate.

To assess cell proliferation following culture in media containing different serum sources, MSCs from three individual horse donors were plated at 100,000 cells/well on 6-well plates in growth media containing either 10% FBS or autologous or allogeneic equine serum. Cells were trypsinized and counted at 24, 48, and 72 h following plating using an automated cell counter (Nexcelom; Bioscience Cellometer Auto T4). Population doubling time was calculated for each of three cell lines cultured in different serum sources over 72 h, as previously reported (47). Experiments were repeated in duplicate for all three donor horses, each in triplicate.

Morphology of MSCs plated in different serum sources (10% serum, either FBS or autologous or allogeneic equine serum) at 100,000 cells/well on 6-well plates was documented and assessed over 72 h. Images were obtained of cells in culture using imaging software (Olympus; CellSens, Tokyo, Japan).

### Cytokine Secretion

Equine MSCs from three donor horses were cultured in growth media with either 10% FBS or autologous or allogeneic equine serum for 72 h and then plated at 100,000 cells/well for 24 h on 24-well plates in media containing their respective serum sources. Supernatants were collected at 24 h and fluorescent bead–based multiplex assay (Milliplex MAP Equine Cytokine/Chemokine Magnetic Beads Multiplex Assay, Millipore Sigma, Burlington, MA) was used to quantify the concentrations of 23 analytes [eotaxin/CCL11, fibroblast growth factor 2 (FGF-2)]. Fractalkine/CS3CL1, granulocyte colony-stimulating factor (G-CSF), granulocyte–macrophage CSF (GM-CSF), growth-regulated oncogene (GRO), interferon (IFN), interleukin 1α (IL-1α), IL-1β, IL-2, IL-4, IL-5, IL-6, IL-8/CXCL8, IL-10, IL-12, IL-13, IL-17a, IL-18, IP-10, monocyte chemoattractant protein 1 (MCP-1), RANTES/CCL5, and tumor necrosis factor (TNF-α) in conditioned media.

### Antimicrobial Peptide (Cathelicidin/LL37) Expression

Equine MSCs from three donor horses were maintained in flasks in growth media containing either 10% FBS or autologous or allogeneic equine serum for 72 h. Cells were then trypsinized, counted, and either stimulated or not with toll-like receptor ligand 3 polyinosinic:polycytidylic acid (pIC) at 10 μg/mL in DMEM media at 2 × 10^6^ cells/mL as previously reported (48) and then plated at 100,000 cells/well on 24-well plates for 24 h in media containing their respective serum sources at either 10 or 1%. Conditioned media was collected and assessed for quantitative antimicrobial peptide cathelicidin/LL-37 by competitive enzyme-linked immunosorbent assay (ELISA), previously validated for expression in human samples and cross-reactive to equine (Human LL37/Cathelicidin Sandwich ELISA kit, Lifespan Biosciences, Seattle, WA), to determine relative levels in conditioned media following activation or resting in different serum concentrations from different sources.

### Bacterial Killing Activity

Equine MSCs form three donor horses were maintained in flasks in growth media containing either 10% FBS or autologous or allogeneic equine serum for 72 h. Cells were then trypsinized, counted, and plated at 100,000 cells/well on 24-well plates for 24 h in media containing their respective serum sources (10%). MSC-CM was collected at that time and frozen at −80°C for use in bacterial killing assays.

The human multidrug-resistant strain of *Staphylococcus aureus* (USA300) was kindly provided by H. Schweizer (Colorado State University). The bacterial culture and sensitivity of this isolate are supplied in Supplementary Document 1. Bacteria were expanded in Luria–Bertani (LB) broth (BD Falcon) and frozen in 20% glycerol until further use. Overnight bacterial cultures were grown in MSC growth media (10% FBS) without antibiotics prior to use in assays. On the day of the bacterial killing assay experiment, bacterial subcultures were grown to log phase in MSC media [OD600 of 0.6, corresponding to 7.5 log10 colony-forming units (CFU/mL)] and then used immediately.

To determine if serum source in cell culture impacted the ability of MSC-CM to directly kill bacteria, MSC-CM from the three horse donors cultured in different serum sources (10%) was inoculated with actively dividing log phase Methicillin-resistant Staphylococcus aureus (MRSA). The antibiotic-free MSC-CM or antibiotic-free MSC growth media (positive control) was plated at 200 μL/well on a 96-well plate, and 500 bacteria in log phase (OD 0.6) were added per well. Coculture plates were incubated shaking at 100 revolutions/min at 37°C for 16 h. Negative control wells containing antibiotic-free DMEM without bacterial inoculation were also included. Following incubation with bacteria, media were transferred to 1.5-mL conical tubes, vortexed to evenly distribute bacteria, diluted 10-fold, plated on LB agar plates (100 μL/quadrant), and incubated at 37°C for 18 h. CFUs were counted manually. Experiments were performed in duplicate for three horses, each in triplicate.

### Chondrogenic Differentiation

Equine MSCs were assessed for chondrogenic differentiation potential following culture for 72 h in either 10% FBS or autologous or allogeneic equine serum by Alcian blue staining (ThermoFisher Scientific StemPro® Chondrogenesis Differentiation Kit, Waltham, MA). Briefly, following culture in respective serum sources for 72 h, cells were trypsinized and washed in phosphate-buffered saline, and the cell pellets resuspended in chondrogenesis medium [StemPro® osteocyte/chondrocyte differentiation basal medium and StemPro® chondrogenesis supplement (9:1), penicillin (100 U/mL) and streptomycin (100 μg/mL)] to generate a cell solution of 1.6 × 10^6^ cells/100 μL and then seeded in 5-μL droplets on 6-well plates. Cells were maintained on plates for 2 h in 37°C incubator at 5% CO_2_, and then chondrogenesis media was added to the plates to cover the pellets. Media was changed every 3 days, and pellets maintained in culture in the 37°C incubator at 5% CO_2_ for a total of 28 days and then evaluated for Alcian blue staining visually by microscopy (Olympus SC30 microscope, Tokyo, Japan).

### Statistical Analysis

Raw data were plotted and visually assessed for normality prior to statistical analysis. Data from the cell proliferation assay were log transformed to improve raw distribution and model fit, whereas other data were judged to be normally distributed. Data were modeled individually using a linear mixed model [function lmer from the lme4 (49) and lmerTest (50) packages] with donor as a random effect to account for differences in donor cell lines. For the bacterial killing assay, proliferation was modeled as doubling time, and for the multiple cytokine secretion assays, media type was modeled as the sole fixed effect. For the antimicrobial peptide expression assay, type of media, pIC activation status, and media concentration were modeled with a 3-way interaction between factors in order to allow for a difference in slope of the fitted line at different media percentages and activation states. The difference in cathelicidin/LL-37 expression was then determined at each of the four combinations of pIC activation (yes/no) and media percentage (1 or 10%).

For both the cell viability assay and proliferation determined by automated cell count, the model was fitted with the fixed effect of media type, time as a continuous factor, and a type–time interaction. The cell proliferation data were log transformed to improve model fit as previously noted. Differences between media types were then evaluated at each time point using estimated marginal means.

Model assumptions of homoscedasticity and normality of error distribution were verified by analysis of QQ plots and fitted vs. residual values, and model fit was judged to be appropriate. Differences between groups were evaluated using differences in estimated marginal means [function emmeans from the emmeans (51) package], with *p*-values adjusted using Tukey adjustment for multiple comparisons or Dunnett test where appropriate. All statistical analyses were performed using R for Mac (R version 4.0.0 “Arbor Day” and 3.6.0 “Planting of a Tree”) (52). Graphical analyses and graph generation were performed using Prism software v8.4.1 (GraphPad Software Inc., La Jolla, CA). For all analyses, statistical significance was assessed as *p* < 0.05.

## Results

### Cell Viability, Proliferation, and Morphology

**Figure 2:**
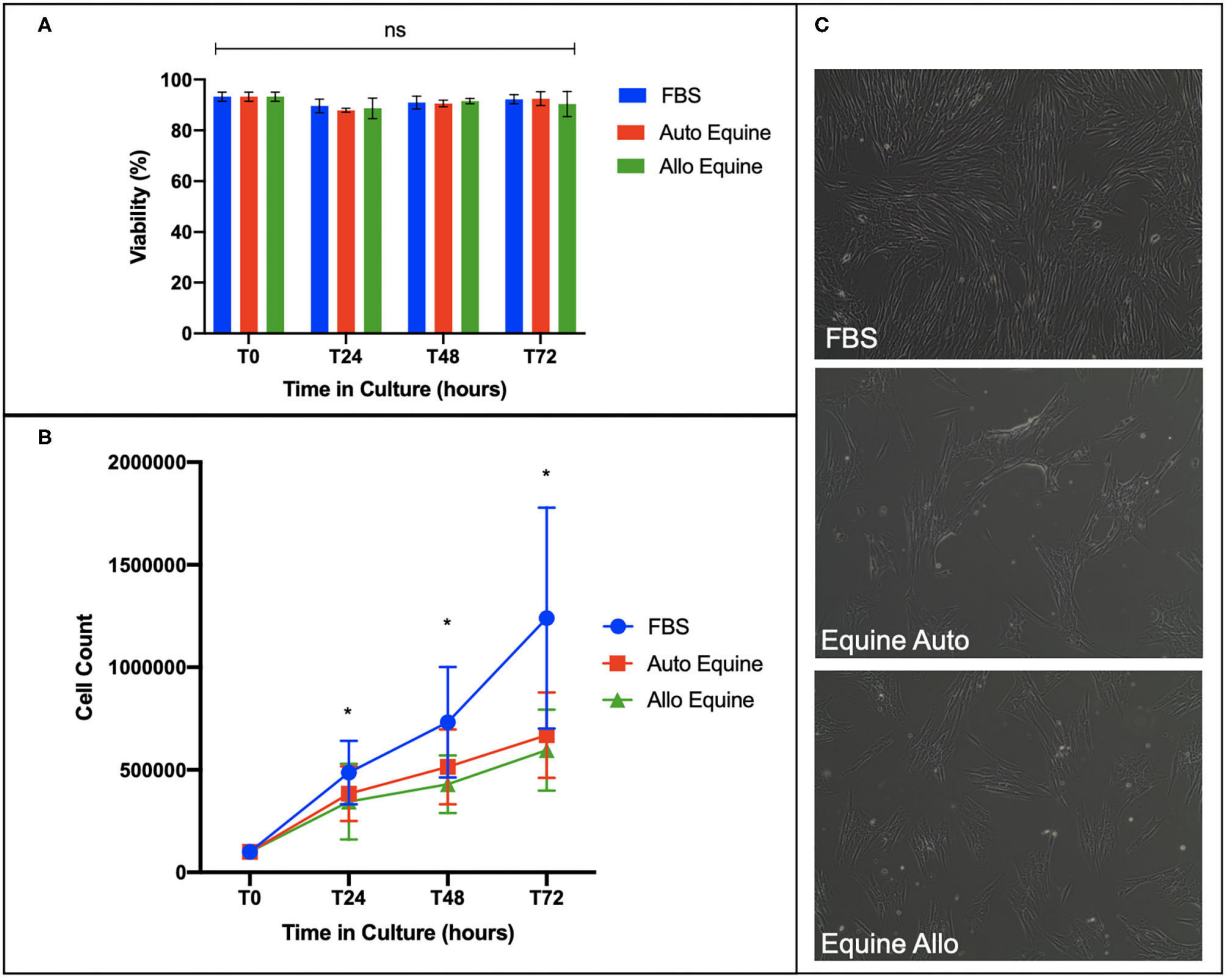
Effect of serum source on cell viability, proliferation, and morphology of equine MSCs in culture. **(A)** Viability of equine MSCs plated at 50,000 cells/well on a 24-well plate in either 10% FBS or autologous or allogeneic equine serum was assessed via trypan blue dye exclusion over 72 h in culture, demonstrating no difference in viability between culture conditions. **(B)** Proliferation of MSCs plated on 6-well plates at 100,000 cells/well was assessed by quantitative cell count at baseline and over 72 h in culture, demonstrating that cells cultured in 10% FBS proliferated at a faster rate compared to those in either autologous and allogeneic equine serum. **(C)** Morphology of MSCs plated in different serum sources on 6-well plates at 100,000 cells/well was assessed visually via microscopy over 72 h. All MSCs demonstrated characteristic fibroblastic morphology, although cells cultured in FBS proliferated faster by visual assessment. Bars are mean and standard deviation of three biological replicates. *Statistical significance assessed at *p* < 0.05. ns, non-significant statistical analysis.

**Figure 3:**
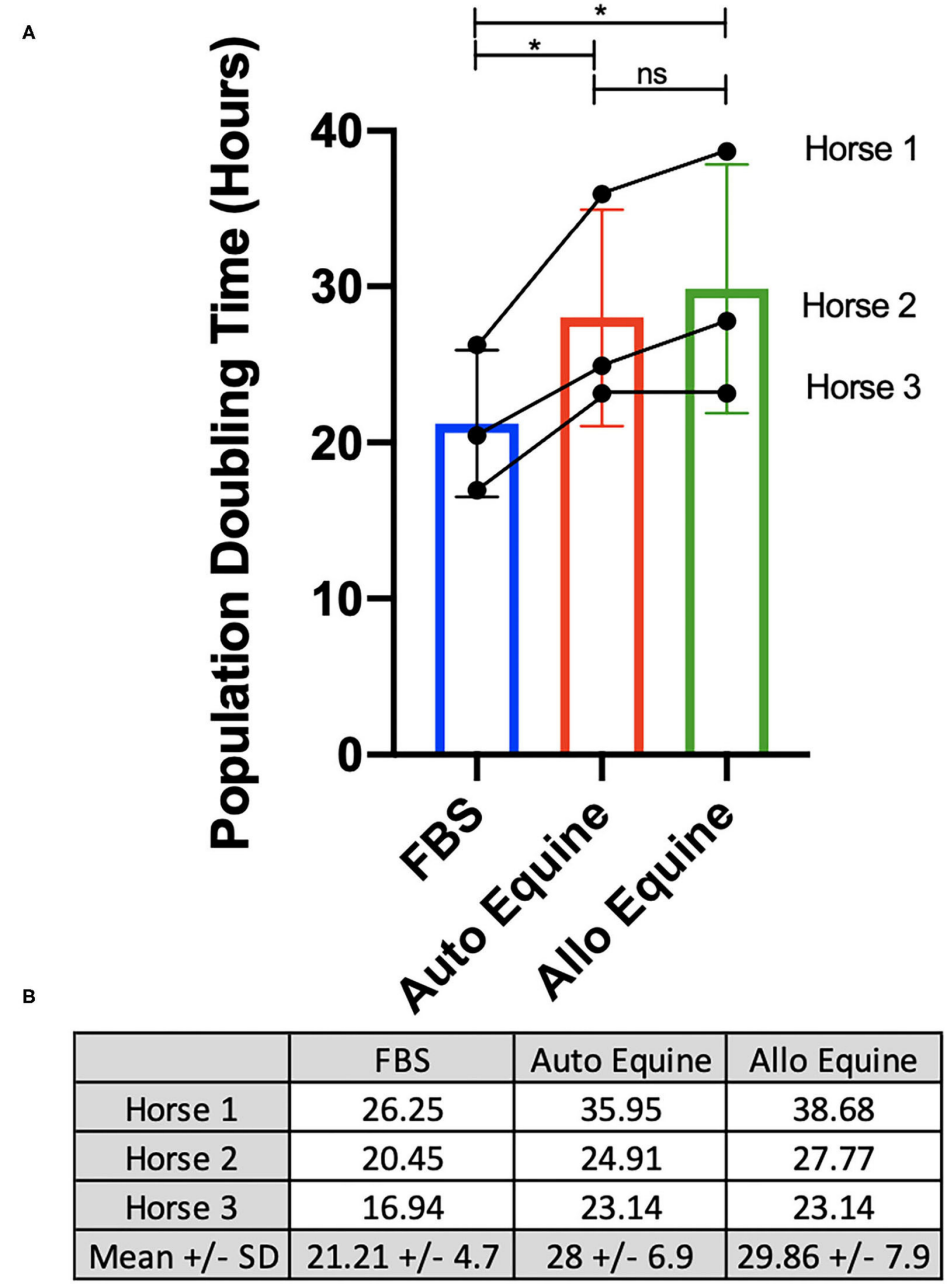
Determination of population doubling time (hours). MSCs from each of three donors (*n* = 3) were plated at 100,000 cells/well on 6-well plates and cultured in different serum sources for 72 h. Population doubling time was determined for each cell line individually and overall, as described in *Methods*, and presented graphically **(A)** and numerically **(B)**. *Statistical significance assessed at *p* < 0.05. ns, non-significant statistical analysis.

Viability of MSCs in culture was not affected by serum source (10%) after 24, 48, or 72 h in culture (Figure 2A). However, proliferation of equine MSCs cultured in FBS was faster compared to those cultured in autologous or allogeneic equine serum at both 48 and 72 h (*p* < 0.001) and faster than those cultured in allogeneic (but not autologous) serum at 24 h (*p* = 0.002) (Figure 2B). Population doubling time (mean ± SD, across three MSC cell lines) was determined to be 21.21 ± 4.7 h in FBS, 28 ± 6.9 h in autologous equine serum, and 29.86 ± 7.9 in allogeneic equine serum. Population doubling times for each of the three MSC cell lines are reported in Figure 3. When analyzed via automated cell count, there were a significantly greater number of cells in culture at 48 and 72 h in FBS media compared to autologous and allogeneic equine serum (*p* < 0.001 for all). There was also a greater number of cells cultured in FBS vs. allogeneic serum containing media at 24 h (*p* = 0.002). Examination of equine MSC cultured in different serum sources did not reveal observable differences in cell morphology over 72-h culture, although proliferation as determined by visual assessment was more rapid in cells cultured in FBS. Representative images documenting cell morphology after 72 h in culture are shown in Figure 2C.

### Cytokine Secretion

**Figure 4:**
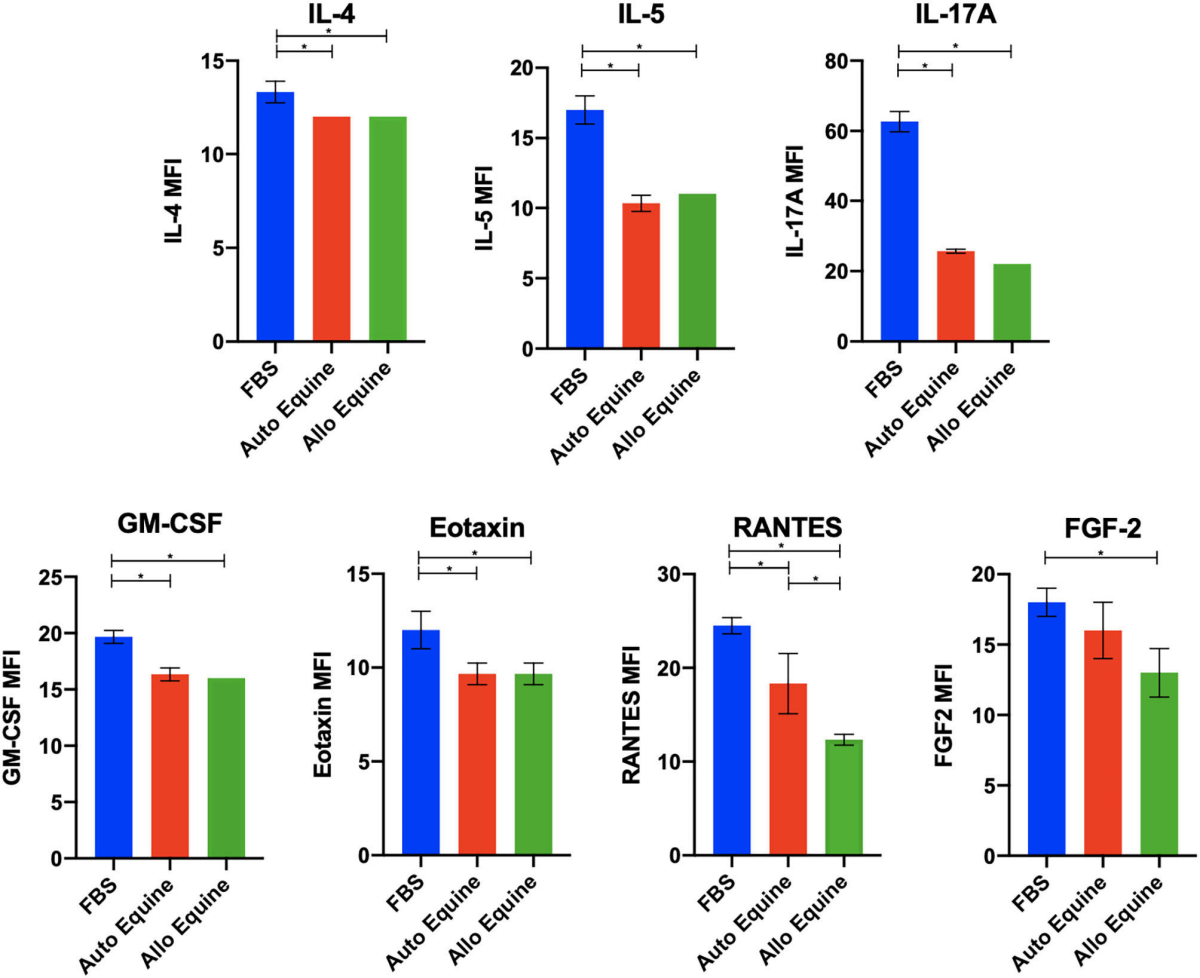
Effect of serum source in cell culture on MSC cytokine secretion. Equine MSCs from three donor horses were cultured in media using different serum sources (10%; FBS or autologous or allogeneic equine serum) for 72 h and then plated for 24 h on 24-well plates at 100,000 cells/well in their respective serum sources. Supernatants were collected after 24 h for evaluation of cytokines on a 23-cytokine fluorescent bead–based multiplex assay. Cells cultured in FBS produced higher levels of seven cytokines (IL-4, IL-5, IL-17A, GM-CSF, eotaxin, RANTES, and FGF-2) compared to autologous or allogeneic serum. Levels of five cytokines were below the detection limit of the multiplex assay (IL-2, IL-12, IL-18, IFN-γ, and MCP-1). For the remaining 11 biomarkers assessed (IL-1a, IL-1B, IL-6, IL-8, IL-10, IL-13, IP-10, TNF-α, GRO, G-CSF, and fractalkine), there were no statistical differences between serum treatment groups in levels of cytokines secreted. Bars are mean and standard deviation of three biological replicates. *Statistical significance assessed at *p* < 0.05.

MSC-CM from cells cultured in FBS (10%) contained higher levels of seven cytokines (IL-4, IL-5, IL-17A, GM-CSF, eotaxin, RANTES, and FGF-2) compared to autologous or allogeneic serum (Figure 4, Tables 1, 2). Levels of five cytokines were below the detection limit of the multiplex assay (IL-2, IL-12, IL-18, IFN-γ, and MCP-1). There were no statistical differences between serum treatment groups for the remaining 11 biomarkers assessed (IL-1α, IL-1β, IL-6, IL-8, IL-10, IL-13, IP-10, TNF-α, GRO, G-CSF, and fractalkine). Cytokine levels in control media were below the detection limit of the multiplex assay.

**Table 1:** Measurable cytokine levels (MFI mean ± SD) in MSC-conditioned media containing either 10% FBS or autologous or allogeneic equine serum.

	**IL-1α**	**IL-1β**	**IL-4**	**IL-5**	**IL-6**	**IL-8**	**IL-10**	**IL-13**	**IL-17A**
FBS	11.3 +/– 1.5	24 +/– 2	13.3 +/– 0.6	17 +/– 1	20.3 +/– 2.5	63.6 +/– 54.5	19 +/– 0	27.3 +/– 21.4	62.6 +/– 2.9
Auto equine	9.8 +/– 0.8	23 +/– 0	12 +/– 0	10.3 +/– 0.6	22.3 +/– 4.5	63 +/– 21.3	20 +/– 1	17.3 +/– 2.3	25 +/– 0.6
Allo equine	10 +/– 0	23.3 +/– 0.6	12 +/– 0	11 +/– 0	24.7 +/– 1.5	59.7 +/– 6.8	20 +/– 1	16 +/– 1	22 +/– 0
**Treatment**	**FGF-2**	**Eotaxin**	**G-CSF**	**GM-CSF**	**Fractalkine**	**IP-10**	**GRO**	**TNF-α**	**RANTES**
FBS	18 +/– 1	12 +/– 1	8.7 +/– 2.1	19.7 +/– 0.6	10 +/– 0	17.3 +/– 2.9	79 +/−107.7	13.3 +/– 0.6	24.5 +/– 0.9
Auto equine	16 +/2	9.6 +/– 0.6	7.6 +/– 0.6	16.3 +/– 0.6	9 +/– 1	15.3 +/– 0.6	8.5 +/– 1.3	16.3 +/– 3.2	18.3 +/– 3.2
Allo equine	13 +/– 1.7	9.6 +/−0.6	7.6 +/– 0.6	16 +/– 0	8.3 +/– 0.6	18.7 +/– 0.6	10.3 +/– 4.2	15.7 +/– 0.6	12.3 +/– 0.6

**Table 2:** Statistical analysis of measurable cytokine levels in MSC-conditioned media containing either 10% FBS or autologous or allogeneic equine serum.

**Treatment**	**IL-1α**		**IL-1β**		**IL-4**		**IL-5**		**IL-6**		**IL-8**		**IL-10**		**IL-13**		**IL-17A**	
FBS vs. auto	ns	0.23	ns	0.59	^**^	0.01	^****^	<0.0001	ns	0.72	ns	0.2	ns	0.36	ns	0.61	^****^	<0.0001
FBS vs. Allo	ns	0.3	ns	0.78	^**^	0.01	^****^	<0.0001	ns	0.28	ns	0.16	ns	0.36	ns	0.54	^****^	<0.0001
Auto vs. Allo	ns	0.98	ns	0.93	ns	>0.99	ns	0.48	ns	0.65	ns	0.98	ns	>0.99	ns	0.99	ns	0.09
**Treatment**	**FGF-2**		**Eotaxin**		**G-CSF**		**GM-CSF**		**Fractalkine**		**IP-10**		**GRO**		**TNF-α**		**RANTES**	
FBS vs. auto	ns	0.36	^*^	0.02	ns	0.63	^***^	0.0003	ns	0.24	ns	0.39	ns	0.41	ns	0.21	^*^	0.02
FBS vs. Allo	^*^	0.02	^*^	0.02	ns	0.63	^***^	0.0002	ns	0.06	ns	0.64	ns	0.42	ns	0.36	^***^	0.0006
Auto vs. Allo	ns	0.14	ns	>0.99	ns	>0.99	ns	0.68	ns	0.49	ns	0.12	ns	0.99	ns	0.91	^*^	0.02

*Statistical significance was indicated as*

* *P ≤ 05*,

** *P ≤ 01*,

*** *P ≤ 001*,

**** *P ≤ 0001*.

### Cathelicidin/LL37 Secretion

**Figure 5:**
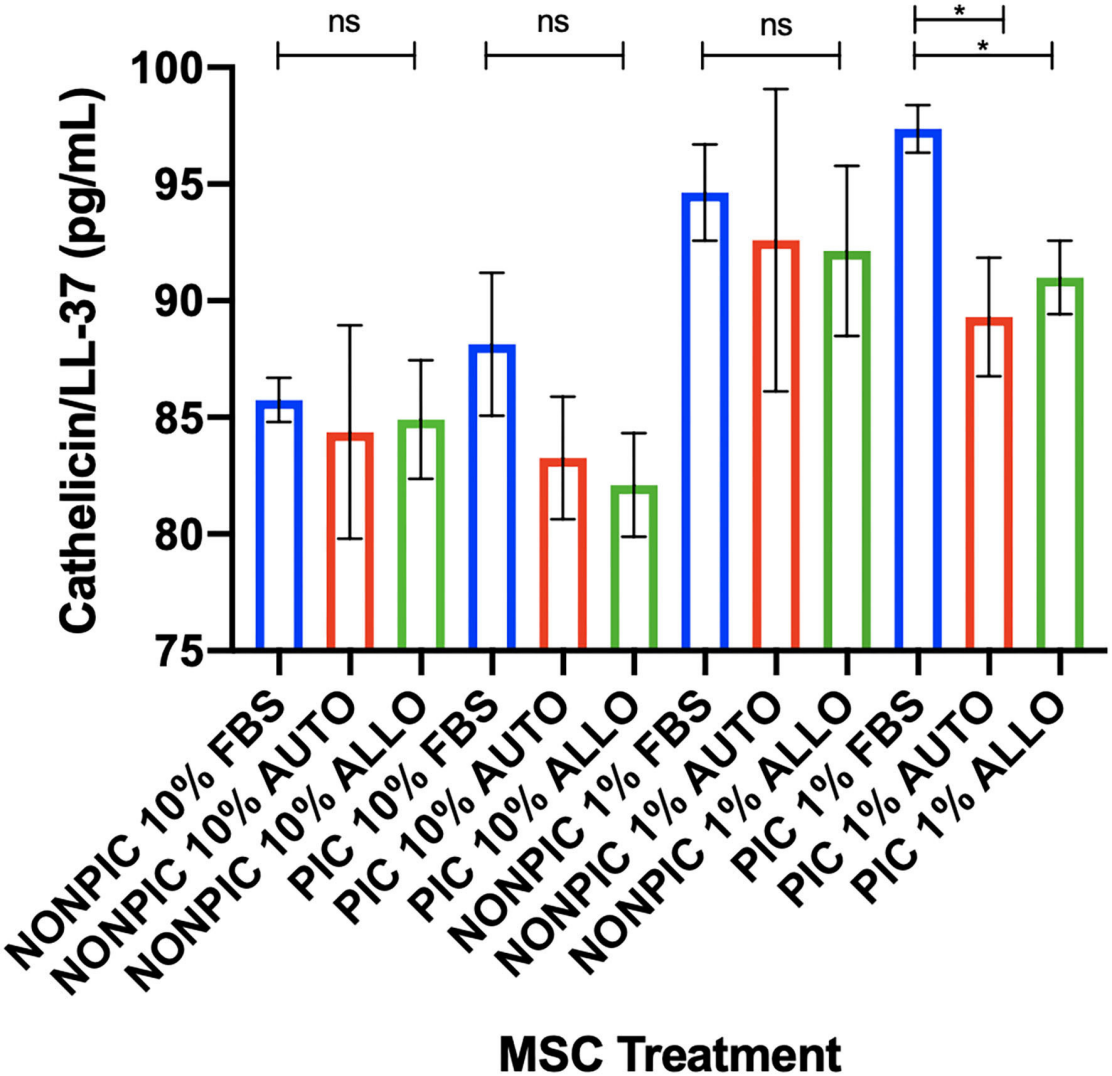
Effect of TLR agonist priming and serum concentration in culture on antimicrobial peptide secretion. Equine MSCs were plated on 75-cm^2^ plates for 72 h in different serum sources (FBS or autologous or allogeneic equine serum) and then trypsinized and stimulated with pIC (10 μg/mL, 2 h, 2 × 10^6^ cells/mL) and plated for 24 h in DMEM media supplemented with varying concentrations (10 or 1%) of different serum sources (FBS or autologous or allogeneic equine serum) on 24-well plates at 100,000 cells/well. Conditioned medium was collected after 24 h in culture and assessed for quantitative cathelicidin/LL-37 antimicrobial peptide production via ELISA. In 1% serum culture following pIC activation, LL37 production was significantly higher from MSCs in FBS cultured media compared to autologous or allogeneic equine serum. Bars are mean and standard deviation of three biological replicates. ns, non-significant statistical analysis. *Statistical significance assessed at *p* < 0.05.

Antimicrobial peptide (cathelicidin/LL-37) expression was higher in MSC-CM from pIC-activated cells cultured in 1% FBS compared to those cultured in autologous or allogeneic equine serum (FBS vs. autologous, *p* = 0.01; FBS vs. allogeneic, *p* = 0.04). No differences in cathelicidin/LL-37 secretion were found between serum treatment groups for cells cultured in either 10 or 1% non–pIC-activated or 10% pIC-activated culture conditions (Figure 5).

### Bacterial Killing Activity

**Figure 6:**
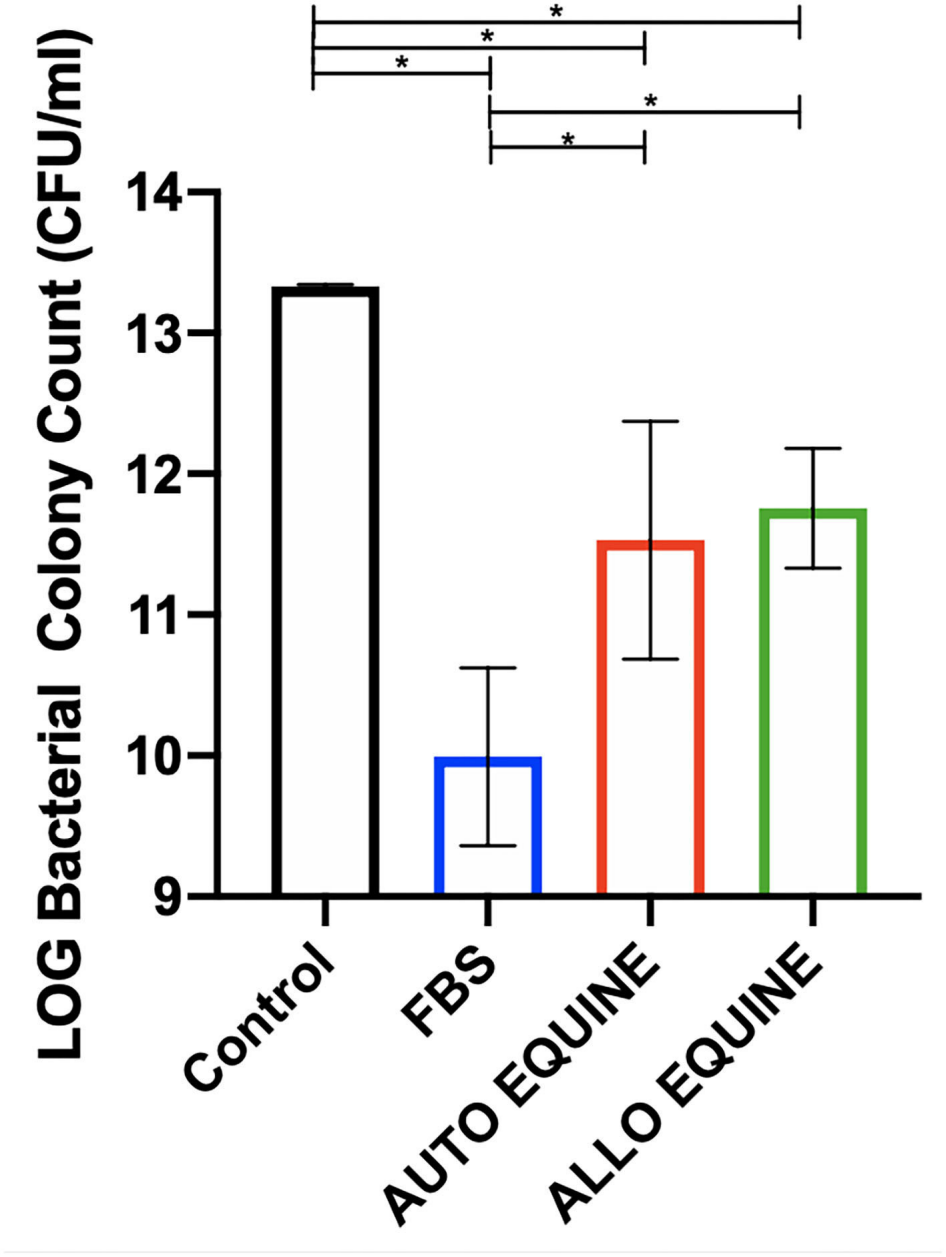
Effect of serum source in culture media on bactericidal activity of MSC-CM on multidrug-resistant *S. aureus*. Multidrug-resistant *S. aureus* was cultured with MSC-CM cultured in media with different serum sources (10% FBS or autologous or allogeneic equine serum) for 16 h and plated on LB agar quadrant plates for 18 h. Bacterial growth shown on *y*-axis was measured by plating bacteria and counting viable colonies. Bacterial growth was reduced by all MSC-CM treatments compared to control and were also further reduced in MSC-CM from FBS-cultured cells compared to autologous or allogeneic equine serum. Bars are mean and standard deviation of three biological replicates. *Statistical significance assessed at *p* < 0.05.

Bacterial growth was inhibited by the MSC-CM cultured in all serum sources compared to control (FBS, *p* = 0.001; autologous, *p* = 0.01; allogeneic, *p* = 0.02) and to the greatest extent with FBS-cultured cells. Bacterial growth was further inhibited by the MSC-CM from FBS-cultured cells compared to those in autologous (*p* = 0.02) and allogeneic equine serum (*p* = 0.04). No differences in bacterial inhibition were seen between autologous or allogeneic MSC-CM treatments (*p* = 0.94) (Figure 6).

### Chondrogenic Differentiation and Gene Expression

**Figure 7:**
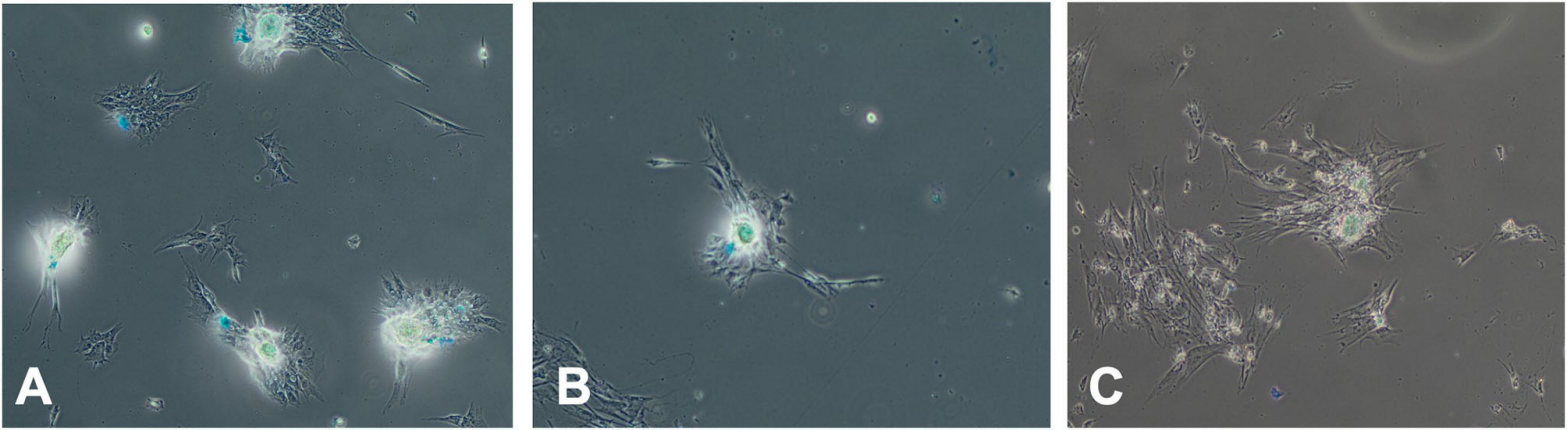
Effect of serum source in culture on chondrogenesis differentiation of equine MSC. Equine MSCs were cultured in 75-cm^2^ flasks for 72 h in media containing either 10% FBS or autologous or allogeneic equine serum and then trypsinized and replated in chondrogenesis differentiation media for 28 days. Media was changed every 3 days. At that time, cells were examined for Alcian blue expression indicating chondrogenic differentiation via microscopy. Serum source [**(A)** FBS, **(B)** autologous equine serum, or **(C)** allogeneic equine serum] for 72 h did not inhibit chondrogenic differentiation.

Chondrogenic differentiation was not inhibited by treatment with media with different serum sources for 72 h prior to differentiation as evidenced by Alcian blue expression at 28 days (Figure 7).

## Discussion

MSCs are increasingly used in equine clinical practice, particularly in the treatment of musculoskeletal disorders, although more recent literature has demonstrated their potential efficacy in other disease processes such as immune-mediated disorders and infection (1–12). Cell culture in defined, serum-free medium has been proposed as an alternative to FBS to reduce antigenicity associated with the proteins themselves and potentially increase longevity of MSCs following injection, which may improve treatment effect (44). However, serum-free medium as an alternative is cost-prohibitive in some situations for routine clinical use. Little is known, however, regarding the relative merits of each serum source with respect to MSC function. In our study, we found that FBS culture resulted in faster proliferation with shorter population doubling times and significantly greater secretion of key cytokines and antimicrobial peptides, which may be relevant to stromal cell function following clinical application in inflammation or infection. These findings should prompt equine practitioners using MSCs in their clinical practice to weigh the relative benefits of different serum sources in cell culture prior to administration.

In this study, we reported that equine MSC viability was not reduced following 72 h in culture with different serum sources, although proliferation was significantly faster, resulting in a greater number of cells in FBS vs. equine serum culture. Chondrogenic differentiation was not suppressed by culture in alternative serum sources to FBS, although full trilineage differentiation was not explored. Previous studies have reported differing results in terms of MSC morphology and capacity for proliferation and differentiation following culture in different media, which may be species dependent (13, 38, 43). For instance, culture of equine MSCs in serum-free conditions was demonstrated to result in expression of a more variable surface phenotype in equine MSCs with lower percentages of CD90^+^ cells and small subpopulations of CD14^+^, CD34^+^, CD45^+^, or MHCII^+^ cells (13). In contrast, human MSCs maintained a more consistent morphology with CD29^+^, CD90^+^, and CD105^+^ immunophenotyped following serum-free culture (13). Furthermore, morphology of equine but not human MSCs was altered with increased detachment of multilayers and cellular aggregation noted (13). These studies, taken together, highlight that variability in cell response to serum-free culture in terms of viability, proliferation, differentiation, and morphology depends on a number of factors and, importantly, should be optimized for the species of interest.

Concentrations of seven biomarkers (IL-4, IL-5, IL-17A, GM-CSF, eotaxin, RANTES, FGF-2) were found to be increased in supernatants collected from MSCs cultured with FBS vs. autologous or allogeneic equine serum. Lack of statistical significance in levels of several other cytokines (e.g., IL-8, IL-13, GRO) may be explained in part due to small number of horse cell lines (*n* = 3) and the large variability between cell lines evaluated. Various studies have demonstrated therapeutic potential for IL-4 in osteoarthritis (OA) (53–58). IL-4 receptors are expressed on chondrocytes and synovial cells (54, 55), and IL-4 signaling has been shown to alter mechanotransduction in chondrocytes associated with turnover of matrix in OA (56). In addition, IL-4 inhibits chondrocyte apoptosis and cartilage breakdown and reduces synovial inflammation by antagonizing TNF-α-induced production of prostaglandin E_2_ (PGE_2_) by synovial fibroblasts in OA (59). Furthermore, genetic variation in IL-4R genes increase susceptibility of individuals to OA (57). Finally, IL-4 induces production of IL-1 receptor antagonist, which may have important implications when MSCs are injected in treatment of OA where IL-1 is frequently elevated (60).

Several of the other cytokines elevated in FBS-cultured MSC-CM have also been implicated to have prognostic value in the degree of severity and progression of OA. Significant concentration differences in IL-5 in synovial fluid were noted between subjects with little or no arthritis compared to those with advanced arthritis based on the ICRS scale (61). When synovial fluid samples from patients undergoing knee arthroscopy were evaluated for biomarker levels, RANTES (regulated upon activation, normal T cell expressed and secreted), in addition to vascular endothelial growth factor and platelet-derived growth factor, was one of the strongest predictors of postoperative improvement at final follow-up regardless of the degree of cartilage injury at time of surgery (62). RANTES has also been reported as a mediator of acute and chronic inflammation, with recruitment of macrophages, mast cells, and eosinophils, as well as PGE_2_ generation demonstrated following RANTES injection in skin of experimental rodent models (63). IL-17 promotes recruitment of both neutrophils and monocytes by inducing various chemokines (64), which has been suggested to mediate inflammation in human rheumatoid arthritis (RA) (65).

Interestingly, FGF-2 has been suggested to have both a chondroprotective role in cartilage metabolism and a catabolic effect on articular cartilage homeostasis and may play a role in cartilage regeneration and repair (66–68). The conflicting roles of FGF-2 have been suggested to be dependent on differences in the balance of FGF receptors (FGFRs) within the tissue of interest, which may be species-dependent (68, 69). For example, FGF-2 induces catabolic and antianabolic effects in human articular cartilage (67, 68) but exerts an overall anabolic effect in murine cartilage (66). At the time of this writing, the reasons for species specificity remain unknown but may potentially be accounted for by differences in receptor levels between species and may be further clarified when species-specific FGFR profiles, including equine, are published (69). Increased eotaxin levels in the synovial fluid of individuals with OA have been reported (70) and correlated with disease severity (71). Eotaxin production by chondrocytes was further demonstrated to be induced by stimulation of chondrocytes with IL-1B or TNF-α (70). In contrast, however, high serum levels of eotaxin are associated with less radiographic progression in early RA patients, suggesting a counter-regulatory role (72). Finally, GM-CSF, elevated in MSC-CM of FBS-cultured stromal cells, has been described as a growth factor that induces proliferation and differentiation of bone marrow myeloid progenitor cells and therefore may exert an important effect to encourage migration of myeloid cells in inflammation, stimulating renewal of macrophages and granulocytes and survival of targeted cells (73). Both protective and pathogenic roles for GM-CSF in inflammatory and autoimmune diseases have been described, demonstrating multiple roles for GM-CSF and potential therapeutic strategies that may exploit its role in inflammation (73).

Equine MSCs possess antimicrobial and immunomodulatory properties, and their application in the treatment of bacterial infections is gaining increasing attention (3, 4). MSCs are normal participants in tissue repair processes, promoting healing through epithelialization, angiogenesis, granulation tissue formation, collagen deposition, and release of inflammatory mediators (74–84). Direct antibacterial effects mediated by secretion of antimicrobial peptides such as cathelicidin/LL-37 (74, 75) and indirect effects through activation and recruitment of immune effector cells as MSCs express genes for production of immunomodulatory and chemoattractant cytokines including IL-6, IL-8, and MCP-1 (74–76, 83, 85). In this study, we demonstrated that cathelicidin/LL-37 production and bactericidal ability to *S. aureus in vitro* was reduced in MSC-CM from cells cultured in autologous or allogeneic equine serum compared to FBS. These findings may have important implications toward optimizing MSC antibacterial activity when used to treat infections, and the relative antimicrobial capacity of equine MSCs cultured in different serum sources warrants further investigation *in vivo*.

Limitations of this study include the *in vitro* nature of design, relatively low number of individual equine cell lines assessed (*n* = 3), short period of time in culture with alternate serum sources (72 h), and the fact the all MSCs were initially cultured in the presence of FBS. Culture of MSCs in their respective media from the beginning of the study could have yielded additional information. It is also acknowledged as a limitation that conditions for MSCs cultured in autologous or allogeneic equine sera were changed vs. those in FBS that remained the same throughout the study. However, the study was performed as such to replicate clinical scenarios where equine practitioners replace the FBS as the serum source several days prior to clinical application. In addition, further *in vivo* evaluation and comparison of safety and efficacy of MSCs that were cultured in different serum sources are warranted. Secretion of additional various cytokines and growth factors that may play a role in joint inflammation and degeneration where MSCs are applied in the treatment of OA such as transforming growth factor β, platelet-derived growth factor, or aggrecanases was not quantified in this study. Additional immunophenotypic evaluation for surface markers of MSCs and comparison of surface marker expression prior to and following culture in different serum sources could have been performed and may have added additional value to the study design. In assessing chondrogenic differentiation, the time period of 72 h in culture is relatively short prior to 28 days of culture in chondrogenesis differentiation assays, and it is acknowledged that although the chondrogenesis differentiation media was serum-free, a potential effect of different sera sources could have been masked by the length of time in culture following removal from the alternate sera sources investigated. The study did not assess the effect of serum donor age on composition of sera; as FBS has demonstrated differences compared to adult bovine serum, the inclusion of and comparison to fetal horse serum here could have also strengthened study design. Finally, this study was limited in comparing FBS to only autologous or allogeneic equine serum, and further studies may also evaluate alternative xenogene-free options to FBS in culture media including platelet lysate and commercially available serum substitutes. Batch-to-batch variability and transmission of species-specific viruses are also a risk with allogeneic equine serum use in culture (as they are with FBS). Therefore, recombinant products represent the only sustainable alternative to FBS, although the price of commercially available media may be prohibitive in some circumstances.

In conclusion, this study demonstrated that MSCs cultured in medium with FBS were more functionally active than MSCs cultured in equine serum. However, no difference was found in MSCs cultured in autologous serum compared to cells cultured in allogeneic equine serum. To ascertain whether these *in vitro* effects translate to clinically significant differences in MSC efficacy, randomized trials comparing the effectiveness of MSCs cultured in FBS to MSCs cultured in equine serum need to be conducted. Until such trials are completed, it is important to consider serum effects not only on cell viability and proliferation, but also on the intended MSC effector cell functions for the given disease indication when opting for cell serum sources.

## Data Availability Statement

The raw data supporting the conclusions of this article will be made available by the authors, without undue reservation.

## Ethics Statement

The animal study was reviewed and approved by Colorado State University Institutional Animal Care and Use Committee.

## Author Contributions

LP, LC, SD, and LG: study conception and design. LP and LC: acquisition of data. GG, LP, LC, SD, and LG: data analysis and interpretation. LP and GG: drafting of manuscript. All authors contributed to and approved the submitted version of the manuscript.

## Conflict of Interest

The authors declare that the research was conducted in the absence of any commercial or financial relationships that could be construed as a potential conflict of interest.

## Supplementary Material

Supplementary Document 1 Bacterial culture and sensitivity of the human MRSA strain of S. aureus (USA300) isolate used in bacterial killing and biofilm assays, with MIC reported in μsg/mL.
