# Supplementary figures and images for: Impact of Three Different Serum Sources on Functional Properties of Equine Mesenchymal Stromal Cells
Source: Front Vet Sci. 2021 Apr 30;8:634064. doi: 10.3389/fvets.2021.634064 (PMC8119767; doi:10.3389/fvets.2021.634064)

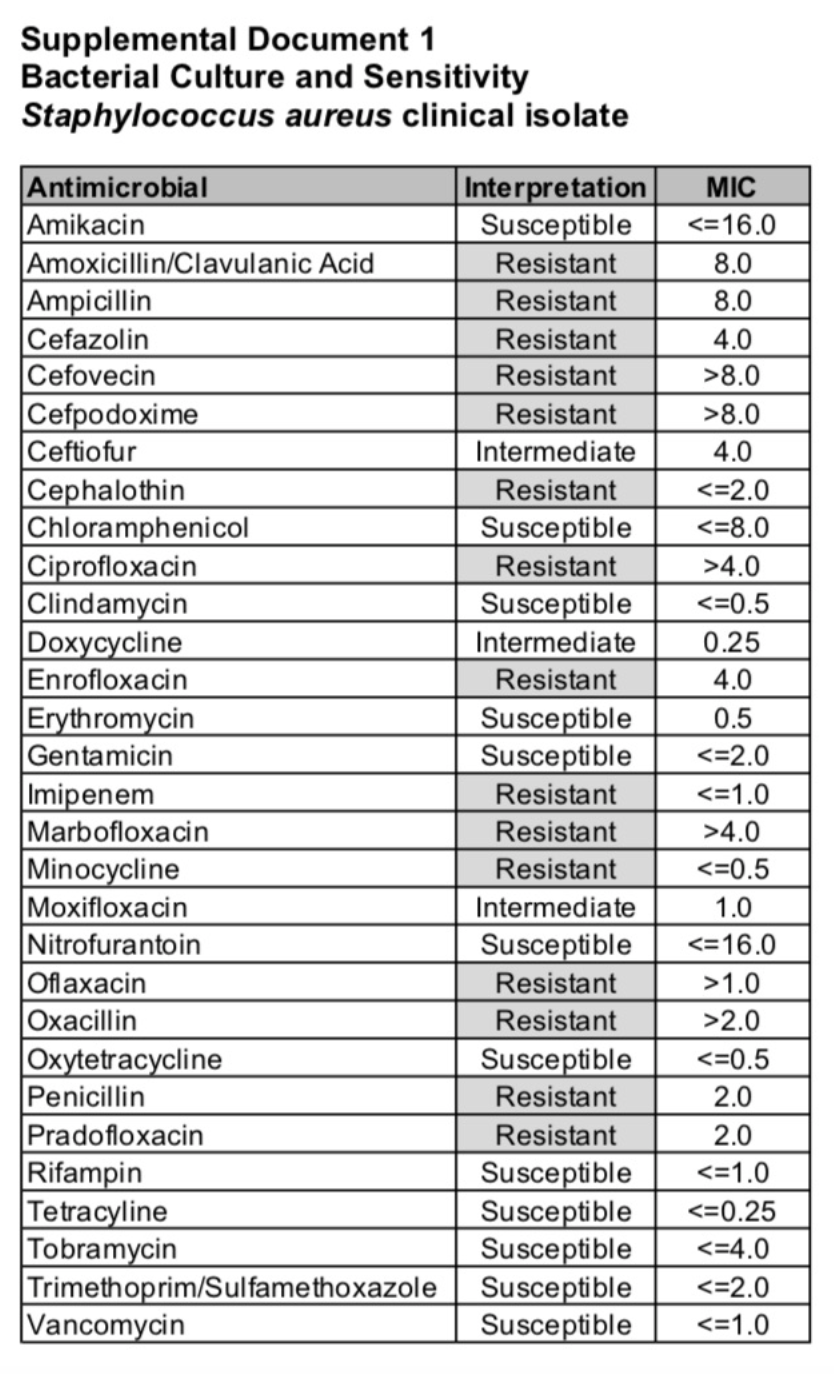

Supplement: Supplementary Document 1 — Bacterial culture and sensitivity of the human MRSA strain of S. aureus (USA300) isolate used in bacterial killing and biofilm assays, with MIC reported in μsg/mL. [file Image_1.TIFF]
